# Supplementary material for: Breaking the angular dispersion limit in thin film optics by ultra-strong light-matter coupling
Source: Nat Commun. 2024 Dec 3;15:10529. doi: 10.1038/s41467-024-54623-1 (PMC11615041; doi:10.1038/s41467-024-54623-1)
Supplement: Supplementary file 1 — Supplementary Information [file 41467_2024_54623_MOESM1_ESM.pdf]

Supplementary Information:

Breaking the angular dispersion limit in thin film optics by ultra-strong light-matter coupling

Andreas Mischok[1,2,*], Bernhard Siegmund[3], Florian Le Roux[1], Sabina Hillebrandt[1,2], Koen Vandewal[3], Malte C. Gather[1,2,*]

[1]Humboldt Centre for Nano- and Biophotonics, Department of Chemistry, University of Cologne, Greinstr. 4-6, 50939 Köln, Germany

[2]School of Physics and Astronomy, University of St. Andrews, North Haugh, St. Andrews KY16 9SS, United Kingdom

[3]Institute for Materials Research (IMO-IMOMEC), Hasselt University, Wetenschapspark 1, Diepenbeek, 3590 Belgium

**contact: andreas.mischok@uni-koeln.de, malte.gather@uni-koeln.de*

Contents

**Supplementary Note 1**	Analysis of metal-organic-metal polariton filters
**Supplementary Figure 1**	Polariton filter transmission for varying Ag mirrors.
**Supplementary Figure 2**	Combination of a polariton-based filter with Spiro-TTB absorption filter
**Supplementary Figure 3**	Angle-resolved transmission for polariton filters with different materials
**Supplementary Figure 4**	Metal-dielectric-metal filters throughout the VIS
**Supplementary Figure 5**	Transmission of MDM and polariton filters with thickness variations
**Supplementary Figure 6**	Transmission of a BSBCz-based filter with different thicknesses
**Supplementary Table 1**	Layer stacks for the optimized DBR-based polariton filters
**Supplementary Note 2**	Analysis of dielectric multilayer filters
**Supplementary Figure 7**	Simulated transmission comparing DBR and Optimized Filter 1 (OPF1)
**Supplementary Figure 8**	Electric field simulation of DBR-based filters
**Supplementary Figure 9**	Transmission of Optimized Filter 2 (OPF2)
**Supplementary Figure 10**	Polarization mixing of polariton-based filters

**Supplementary Note 3**	Performance limits and comparison of polariton filters
**Supplementary Figure 11**	Ag-Ag polariton filters with optimized peak transmission
**Supplementary Figure 12**	Comparing polariton filters against MDM filters, absorptive filters and combined MDM / absorptive filters
**Supplementary Figure 13**	Tunability of polariton filters
**Supplementary Figure 14**	Polarized transmission of polariton filters
**Supplementary Figure 15**	High-performance design of a DBR-based longpass polariton filter
**Supplementary Figure 16**	Literature comparison of angle-stable bandpass transmission filters
**Supplementary Table 2**	Literature comparison of angle-stable bandpass transmission filters
**Supplementary Note 4**	Analysis of the monolithic filter-photodiode device
**Supplementary Figure 17**	Current-density – voltage characteristics of filtered and reference photodiodes.
**Supplementary Figure 18**	Simulations of optical field in polariton-filter-photodiode.

Supplementary Note 1

In the most basic case, a bandpass filter comprise a thin film sandwiched by two reflective surfaces, such as thin metallic mirrors, where the thin film thickness and mirror reflectivity determine the resonance wavelength and finesse of the filter, respectively. In addition, for thin metal mirrors the reflected phase ϕ of light is a function of metal thickness and leads to a significant deviation from the basic resonance condition for such cavities; this effect can be described by an effective cavity thickness[1]: $d_{\mathrm{eff}} = d + \lambda_{\mathrm{res}}/2\pi n \, * (|\phi_{\mathrm{bot}}| + |\phi_{\mathrm{top}}|)$. This is taken into account for the device design of the devices shown in Fig. 2 of the main text.

Correct tuning of the strong coupling parameters is crucial to achieve the best possible performance. As discussed in the main manuscript, ideally polariton filters operate at a large coupling strength $\hbar\Omega_R/2$ (ideally in the ultra-strong coupling regime) and at a moderate positive detuning. A large value of $\hbar\Omega_R/2$ helps in two ways. First, a large separation of upper and lower polariton branches is desirable to split up the two resonances. In a filter application, a single transmissive resonance is often required. When the separation between UPB and LPB is large, it is easy to filter out the unwanted UPB transmission (if necessary) or utilise an additional absorber to reduce it (compare Supplementary Fig. 2, where the UPB from a C545T cavity is absorbed by an additional SpiroTTB layer outside the cavity). Second, a large coupling strength will lead to a larger separation of the LPB from the bare exciton resonance. This avoids parasitic absorption of uncoupled excitons at the LPB resonance and therefore maintains high transmission. Finally, the high coupling strength tends to allow for a flatter angular dispersion, especially if the transmission band is to occur away from the exciton resonance.

Similarly, the positive detuning is useful to keep a largely angle-independent dispersion. For a large negative detuning, the LPB is highly photonic at lower angles and thus retains the strong angular dispersion of the cavity mode. By using a positive detuning, we aim to make the dispersion more excitonic and thus flatter. However, at the same time, the positive detuning should not be made too large as this will reduce the transmission of the LPB. Through optical simulations, we have empirically found a value of 100 meV to 200 meV to be ideal. We showcase the transition from low to high transmission further below.

The full filter stack comprises: 1 nm Al | 25 nm Ag | 80 nm C545T | 1 nm Al | 25 nm Ag, and is fabricated on a glass substrate via thermal evaporation. In comparison, the architecture of the conventional metal-dielectric-metal (MDM) filter is 1 nm Al | 25 nm Ag | 140 nm SiO_2 | 1 nm Al | 25 nm Ag and is fabricated by a combination of thermal evaporation (for metals) and magnetron sputtering (for SiO_2). In order to maximize resonator quality and transmission, the thin silver mirrors are supported by a seed or wetting layer of aluminium, which is known to support growth of smooth silver films with increased optical quality[2]. Resonator quality can be further tuned by adjusting the silver thickness (compare Supplementary Fig. 1).

Supplementary Figure 3 shows the full spectra of the polariton filters shown in Fig. 2h of the main article. In addition, we show simulated transmission spectra for a corresponding set of MDM filters with a SiO_2 spacer, tuned to have transmission peaks for normal incidence at similar wavelengths, in Supplementary Figure 4. For these, the strong angular shift and resulting overlap of the transmission spectra severely limits the usable angular range.

A further advantage of polariton filters over conventional interference filters is their higher resistance to thickness variations which might be introduced by production errors or local inhomogeneities. Supplementary Figure 5 shows the simulated transmission spectra of an MDM filter and a C545T-based polariton filter for a variation in core-layer thickness of ±5%. For this thickness variation, the polariton filter exhibits a 3-fold lower spectral shift than the

MDM filter. In general, polariton filters are tunable by a variation in thickness, however at the cost of angular stability for red-shifted wavelengths and peak transmission for blue-shifted wavelengths. Supplementary Figure 6 demonstrates this trade-off in the measured angle-resolved spectra for a set of BSBCz-based polariton filters with cavity thicknesses between 40 nm and 70 nm. In practice, thickness tuning alone would not be used over such a wide wavelength range; instead the chemical versatility of organic materials allows to choose well-matched absorber materials for the desired spectral response.

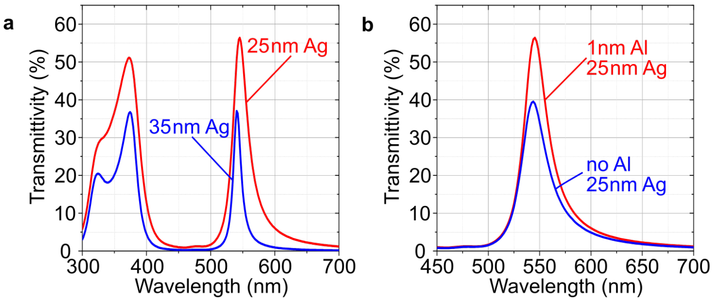

Supplementary Figure 1: Polariton filter transmission for different Ag mirrors. **a** Transmission of a C545T-based polariton filter with Ag mirror of two different thicknesses. Increasing the Ag thickness leads to a narrower linewidth but lower transmission of the lower polariton mode. **b** Transmission of a C545T-based polariton filter with 25 nm thick Ag mirrors, with and without a 1 nm Al seed layer below each Ag layer. The Al seed leads to improved growth of Ag and thus a higher transmission (56% with vs 40% without Al in this example) and a sharper linewidth (27 nm FWHM with and 29 nm FWHM without Al), despite the poor optical properties of the Al layer itself.

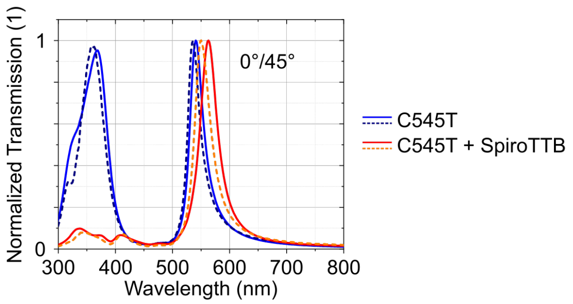

Supplementary Figure 2: Combination of a polariton-based filter with a Spiro-TTB absorption filter. Normalized transmission of polariton filters with the structure 1nm Al | 25nm Ag | 80nm C545T | 1nm Al | 25nm Ag (blue lines) or 1nm Al | 25nm Ag | 90nm C545T | 1nm Al | 25nm Ag | 150nm SpiroTTB (red lines) at 0° (solid lines) and 45° (dashed lines) angle of incidence. The upper polariton branch of the C545T-based polariton filter shows high transmission at 350 nm, but this can be significantly reduced by the additional absorptive layer of Spiro-TTB.

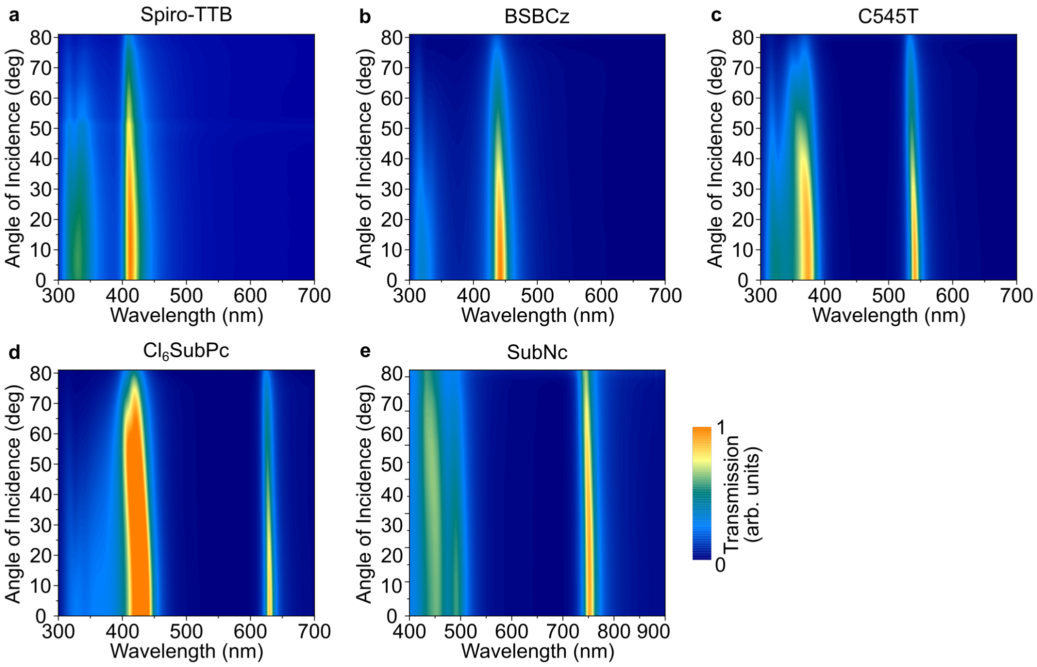

Supplementary Figure 3: Angle-resolved transmission for polariton filters with different materials. Measured angle-resolved transmission of polariton-based filters with the structure: 1 nm Al | 35 nm Ag | SC layer | 1 nm Al | 35 nm Ag, with an optimized thickness of the SC layer tuned to the respective material and desired transmission wavelength: **a** 60 nm Spiro-TTB, **b** 50 nm BSBCz, **c** 90 nm C545T, **d** 80 nm Cl_6SubPc, and **e** 85 nm SubNc. By choosing an appropriate organic material with desired excitonic absorption, polariton filters with narrow and near angle-independent transmission bands across the visible spectral range can be fabricated. Spectra shown here correspond to data shown in Figure 3 of the main manuscript.

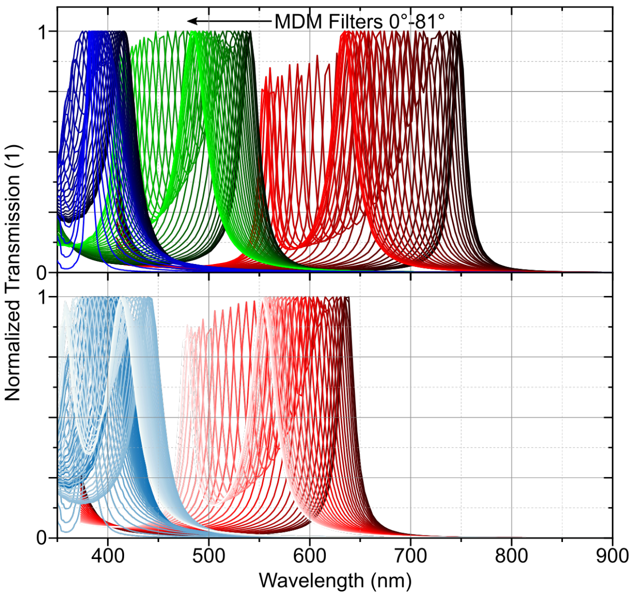

Supplementary Figure 4: Metal-dielectric-metal filters with transmission bands across the visible spectrum. Simulated transmission spectra of MDM filters with the structure 1 nm Al | 35 nm Ag | xx nm SiO_2 | 1 nm Al | 35 nm Ag. The thickness of the SiO_2 layer is adjusted to between 95 nm and 210 nm, to yield peak transmission wavelengths at normal incidence that are similar to the polariton filters presented in Figure 3 of the main manuscript. The spectra of the MDM filters strongly shift with angle, and the spectra of the different filters show strong overlap at oblique angles, limiting their useful angular range.

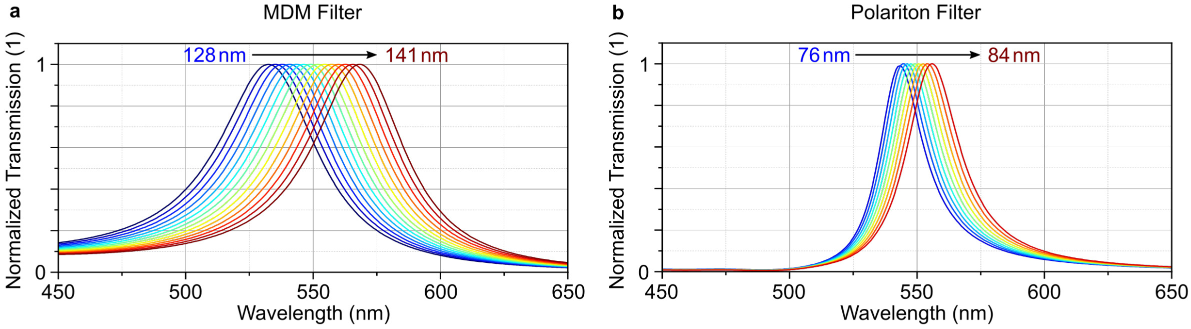

Supplementary Figure 5: Transmission of MDM and polariton filters for different core-layer thicknesses. a Simulated transmission of an MDM filter with the structure 1 nm Al | 25 nm Ag | 135 nm SiO_2 | 1 nm Al | 25 nm Ag and with a thickness variation of the core layer of ±5% (arrow). This thickness variation leads to a spectral shift of 35.6 nm in the resulting bandpass filter. **b** Simulated transmission of a C545T-based polariton filter with the structure 1 nm Al | 25 nm Ag | 80 nm C545T | 1 nm Al | 25 nm Ag and with a thickness variation of the core layer of ±5% (arrow).The polariton filter shows a higher robustness to thickness variation with a spectral shift of only 12.5 nm.

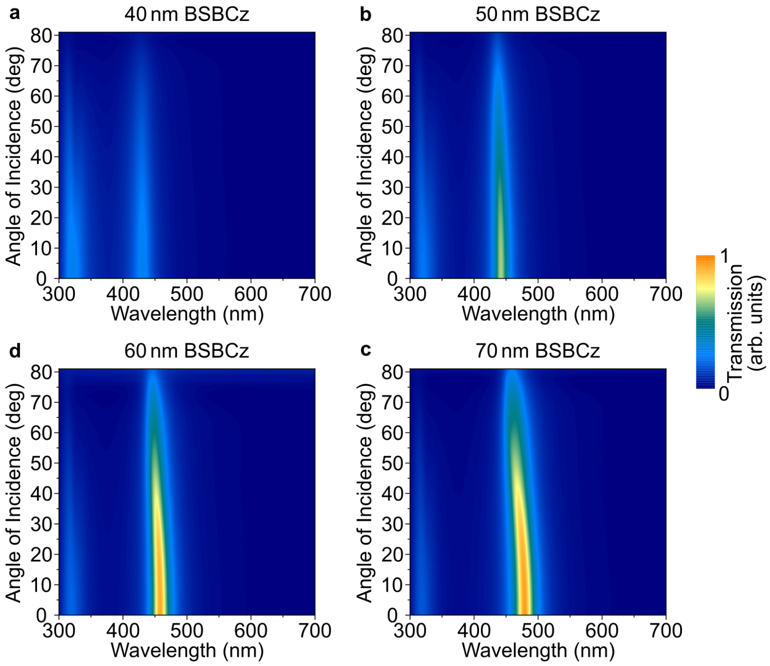

Supplementary Figure 6: Transmission of a BSBCz-based filter with different thicknesses. Measured transmission spectra of BSBCz-based polariton filters with a BSBCz core layer thickness of **a** 40 nm, **b** 50 nm, **c** 60 nm, and **d** 70nm. A thickness variation of the organic core layer allows to tune the spectral position of the transmitted lower polariton branch, however at the cost of reduced peak transmission for blue-shifted resonances and an increased angular dependence for the most red-shifted resonances. In practice, a well-matched absorber material can be selected for the required spectral response, such that the thickness and associated detuning provide an ideal balance between peak transmission and angular stability.

Supplementary Table 1: Layer stacks for the optimized DBR-based polariton filters.

optimized Filter 1 (OPF1)			optimized Filter 2 (OPF2)		
**Layer #**	**Material**	**Thickness (nm)**	**Layer #**	**Material**	**Thickness (nm)**
1	Ta_2O_5	44.5	1	Ta_2O_5	36.6
2	SiO_2	55.0	2	SiO_2	63.5
3	Ta_2O_5	57.8	3	Ta_2O_5	52.2
4	SiO_2	64.1	4	SiO_2	81.2
5	Ta_2O_5	64.1	5	Ta_2O_5	52.9
6	SiO_2	62.3	6	SiO_2	76.3
7	Ta_2O_5	59.7	7	Ta_2O_5	52.2
8	SiO_2	67.6	8	SiO_2	77.3
9	Ta_2O_5	53.0	9	Ta_2O_5	34.8
**10**	**C545T**	**100.0**	**10**	**Spiro:TTB**	**147.0**
11	Ta_2O_5	40.9	11	Ta_2O_5	40.4
12	SiO_2	69.7	12	SiO_2	67.4
13	Ta_2O_5	64.6	13	Ta_2O_5	43.4
14	SiO_2	58.7	14	SiO_2	50.8
15	Ta_2O_5	69.5	15	Ta_2O_5	22.5
16	SiO_2	59.2	16	SiO_2	55.1
17	Ta_2O_5	64.7	17	Ta_2O_5	44.5
18	SiO_2	65.0	18	SiO_2	68.1
19	Ta_2O_5	48.5	19	Ta_2O_5	35.1
**20**	**C545T**	**100.0**	**20**	**Spiro:TTB**	**152.5**
21	Ta_2O_5	55.0	21	Ta_2O_5	37.7
22	SiO_2	71.2	22	SiO_2	75.2
23	Ta_2O_5	62.9	23	Ta_2O_5	48.3
24	SiO_2	60.4	24	SiO_2	65.6
25	Ta_2O_5	69.0	25	Ta_2O_5	36.0
26	SiO_2	61.4	26	SiO_2	22.6
27	Ta_2O_5	62.3	27	Ta_2O_5	41.7
28	SiO_2	61.5	28	SiO_2	64.7
29	Ta_2O_5	47.5	29	Ta_2O_5	54.7
**30**	**C545T**	**100.0**	30	SiO_2	76.5
31	Ta_2O_5	55.8	31	Ta_2O_5	50.5
32	SiO_2	71.1	32	SiO_2	60.0
33	Ta_2O_5	54.0	33	Ta_2O_5	22.1
34	SiO_2	63.2	34	SiO_2	29.0
35	Ta_2O_5	64.6	35	Ta_2O_5	39.9
36	SiO_2	72.0	36	SiO_2	63.2
37	Ta_2O_5	52.7	37	Ta_2O_5	32.4
38	SiO_2	62.2	**38**	**C545T**	**142.6**
39	Ta_2O_5	67.9	39	Ta_2O_5	37.8
40	SiO_2	89.6	40	SiO_2	70.6
41	Ta_2O_5	13.5	41	Ta_2O_5	48.3
			42	SiO_2	68.1
			43	Ta_2O_5	69.9
			44	SiO_2	68.5
			45	Ta_2O_5	47.1
			46	SiO_2	70.3
			47	Ta_2O_5	29.4
			**48**	**C545T**	**147.8**
			49	Ta_2O_5	37.4
			50	SiO_2	61.8
			51	Ta_2O_5	46.5
			52	SiO_2	78.1
			53	Ta_2O_5	56.9
			54	SiO_2	43.1
			55	Ta_2O_5	57.6

Supplementary Note 2

The introduction of organic materials into dielectric filters can lead to significantly enhanced angular performance. Supplementary Figure 7 shows a transfer matrix model simulation that compares a 41-layer DBR to the 41-layer OPF1 from the main text. While the maximum optical density of both filters in the stopband is comparable, the strongly coupled filter shows a much improved angular stability, with a continuous stopband between 400 nm and 500 nm even at extreme angles of incidence. By contrast, the stopband of the conventional DBR shifts completely out of its original position for large angles. While the experimentally achieved performance of OPF1 is slightly decreased relative to the optical simulation (compare main text, Fig. 3**c**), the experimental behaviour is consistent with the simulated performance.

Supplementary Figure 8 further elucidates the angular behaviour for the conventional DBR and for OPF1 and OPF2 using simuations of the electric field distribution inside the filters for light entering from the top side, first at 0° and in a second simulation at 60° angle of incidence. A gradual decrease in field intensity can be seen in the stopband for both the conventional DBR and the OPF1. However, while the stopband shifts drastically for the conventional DBR at an angle of incidence of 60°, the evolution of the electric field is relatively independent of angle in the strongly coupled OPFs.

Supplementary Figure 9 shows the experimentally obtained performance of optimized Filter 2 on glass (Supplementary Fig. 9**a**) and as a freestanding, flexible filter (Supplementary Fig. 9**b**). The performance, in particular the OD, is not compromised by going to the flexible design.

Both metal and dielectric polariton filters show no mixing of polarization. Supplementary Figure 10 shows the lack of cross-polarized sp and ps transmission at 0° and 45° incidence, comfirming no polarizing mixing or scrambling takes place in the polariton-based filters.

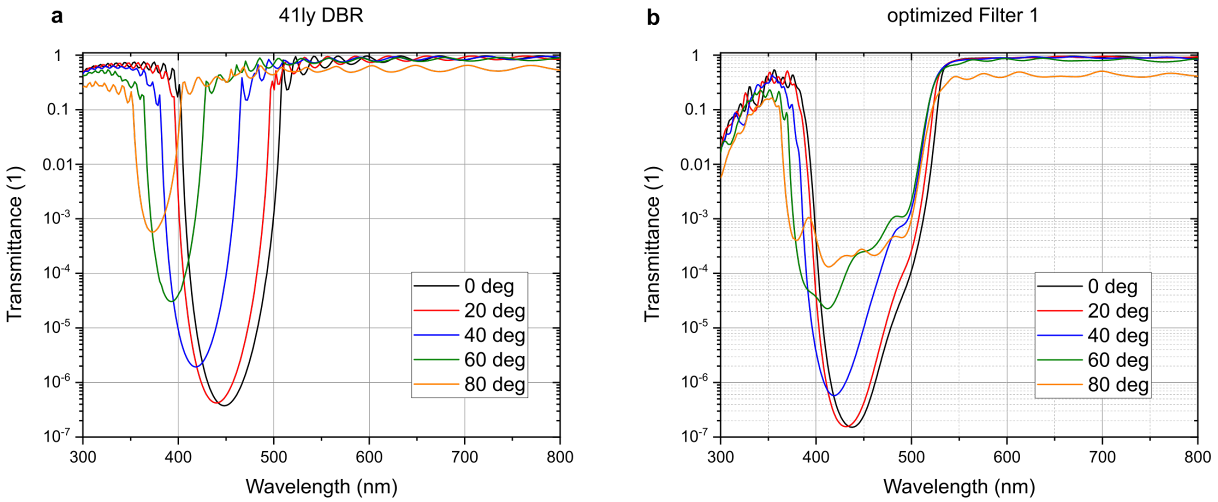

Supplementary Figure 7: Simulated transmission comparing DBR and Optimized Filter 1 (OPF1). **a** Transmittance of a 41-layer conventional DBR at different angles of incidence. **b** Transmittance of OPF1 (also with 41 layers) at different angles of incidence. The performance at normal incidnece is comparable to the conventional DBR but the angular stability of the stopband is significantly increased.

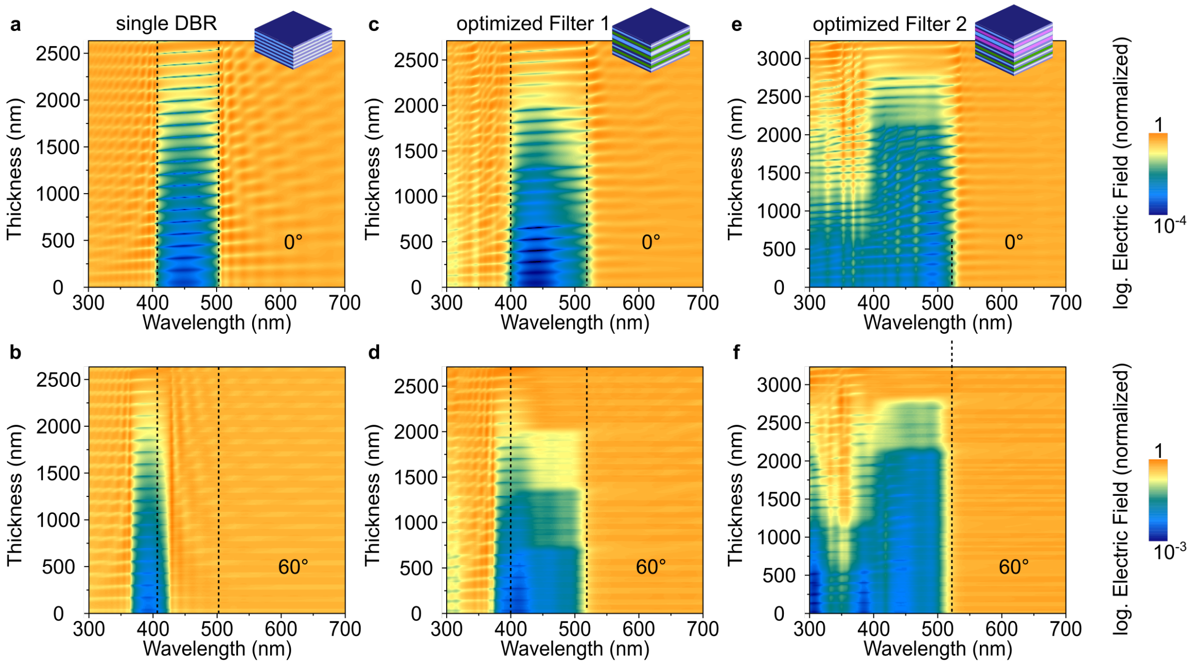

Supplementary Figure 8: Electric field simulation of DBR-based filters. a,b Electric field in a 41-layer conventional DBR with light incident from the top at 0° (**a**) and 60° (**b**) angle of incidence. Dashed lines are a guide to the eye indicating the stopband position. At 60°, the stopband becomes much narrower and almost completely shifts outside of its initial position. **c,d** Electric field in OPF1 (also with 41 layers) with light incident from the top at 0° (**c**) and 60° (**d**) angle of incidence. Utilizing 3 organic cavities with C545T slightly broadens the stopband and renders it largely angle-independent. **e,f** Electric field in OPF2 (with 55 layers) with light incident from the top at 0° (**e**) and 60° (**f**) angle of incidence. The stopband can be broadened further by a stacked DBR design with 2x2 organic cavities of Spiro:TTB and C545T, creating a broadband, angle-independent longpass filter.

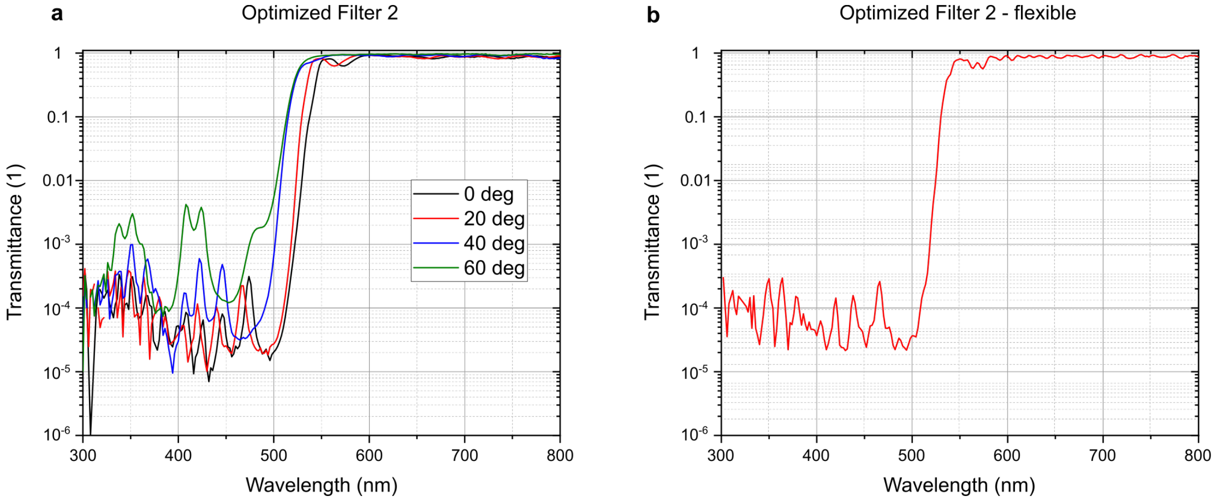

Supplementary Figure 9: Transmission of Optimized Filter 2 (OPF2). a Measured transmittance of OPF2 on logarithmic scale for different angles of incidence. **b** Transmittance of OPF2 in a flexible, substrate-less design using Parylene-C carrier layers.

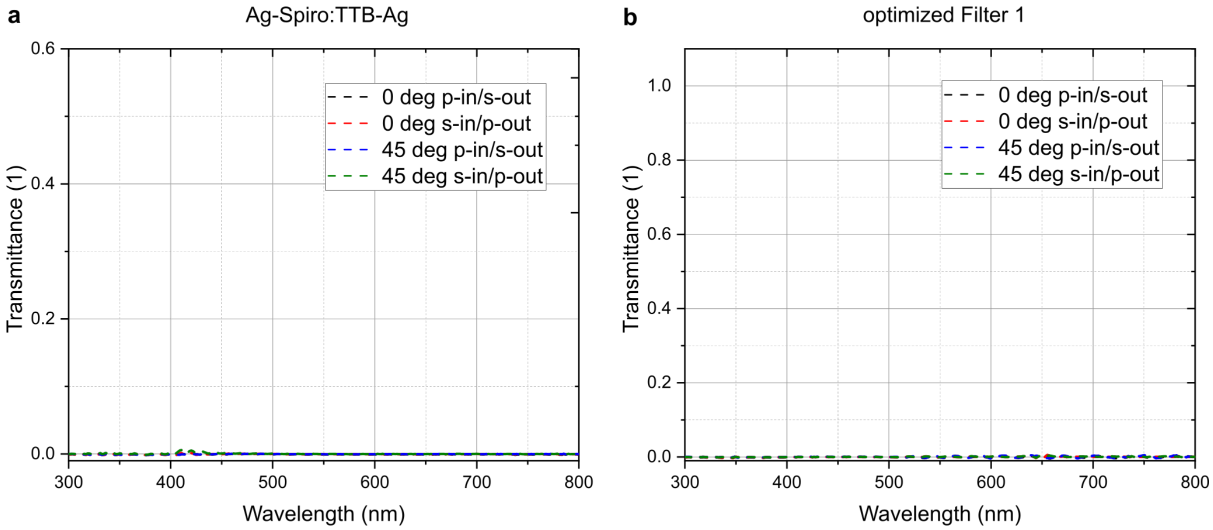

Supplementary Figure 10: Polarization mixing of polariton-based filters. a,b Measured transmission of light with linear cross-polarization (s,p) of incident (in) and detected (out) light when passing through a metal-organic-metal SC filter (**a**) and the DBR-based Optimized Filter 1 (**b**) at 0° and 45° angle of incidence. The transmitted light shows no signs of polarization mixing or scrambling of polarization when passing through the polariton-based filters but instead maintains its linear polarization.

Supplementary Note 3 – Performance limits and comparison of polariton filters

To utilize the advantageous properties of polariton filters, their performance needs to be competitive when compared to the state-of-the-art in conventional optical coatings. In order to fully assess the performance achievable with polariton filters, we performed transfer matrix simulations in order to ensure that the discussion of fundamental limits not to be impacted by losses related to fabrication imperfections – such as enhanced plasmonic absorption by non-ideal silver film quality. Supplementary Figure 11 showcases metal-metal polariton filters with optimized peak transmission, using ideal thin silver films[3] of 25 nm thickness. For such filters the peak transmission can easily reach values above 70% and up to 84%, depending on material, even without further enhancement by the use of anti-reflection layers, which are commonly used in conventional optical coatings.

One might assume that the introduction of organic materials to achieve strong coupling introduces additional absorption losses. However, we find that transfer matrix simulations of conventional MDM filters that use the same silver mirrors as above reach a similar performance (76% peak transmission for a SiO_2 core, 82% for a Ta_2O_5 core). Further analysis indeed shows that the remaining losses in our metal-metal polariton filters originate mainly from residual absorption in the metal layers and from reflection at the outer air-silver interface. Absorption in the organic materials does not represent a significant loss pathway (absorption at peak <3%). When the filter is properly tuned, parasitic absorption from the lower polariton can be almost fully avoided due to its redshift compared to the bare exciton.

Furthermore, the transition from photonic to polaritonic dispersion not only enables a mode with excellent angle stability, it also leads to higher peak transmission at larger angles. Supplementary Fig. 11 **b** shows the maximum transmission of a SubPc-based polariton filter versus angle compared to a conventional MDM filter with a SiO_2 core. The polariton filter maintains a transmission above 75% for angles of incidence up to 50° and above 60% transmission for all angles, while the conventional filter drops to 50% at 50° and 45% above 60°. We believe this behaviour is due to the high refractive index that organic materials show on the red side of their excitonic absorption band because of the *Kramers-Kronig* relation. In this context, we would also like to point out that while the nominal transmission inevitably decreases with increasing angle of incidence for all filters, this effect is more than compensated by the much larger solid angle available at higher angles of incidence. For example, for a beam of light homogeneously covering +/-60° angle of incidence, 73% of the total light intensity is in the 30...60° cone. Therefore, a small drop in transmission at large angles does not significantly diminish the usefulness of having access to these larger angles.

The polariton filters presented here fundamentally differ from a simple combination of an MDM filter and an absorptive filter. For the latter, the blue-shifted transmission at higher angles would simply be absorbed, resulting in a sharp drop in transmission without a remaining detectable mode. Instead, in polariton filters, light is redirected into the polariton branches. In this way, the LPB retains a high and spectrally stable transmission over a much larger angle range, while transmission in the blocking region between LPB and UPB is still strongly suppressed. To demonstrate the difference between a polariton filter and a combination of an MDM structure and an organic absorbing material placed outside MDM cavity, Supplementary Figure 12 shows the angle-resolved transmission of a SubPc-based polariton filter, a conventional MDM filter with SiO_2 as the dielectric, a bare SubPc layer used as a purely absorptive filter, as well as the combination of MDM and organic absorptive filter located outside the MDM cavity; for the latter, we distinguish the case where the organic material is outside but in direct contact with the MDM cavity and the case where the organic materials and the MDM cavity are on opposite sides fo the substrate. This comparison demonstrates

that only the polariton filter shows a strongly improved angular performance, allowing for transmission of light at a stable wavelength even at high angles, while all other filter variants show a strong shift in wavelength with angle, a broad and unspecific transmission spectrum, or a dramatic loss in absolute transmission at larger angles.

While there is an optimal position of the polariton mode to achieve the best possible filter performance for any given material, there is nevertheless significant room to adjust the spectral position of the transmitted mode, while maintaining high transmission and low angular dispersion. Supplementary Figure 13 explores the tunability of an optimized SubPc-based polariton filter, changing the core layer thickness from 50 nm to 100 nm. In turn, the peak transmitted wavelength shifts by ~80 nm. While at low thickness, i.e. for severely blue-shifted cavities, the transmission is drastically reduced, we observe a stable high transmission for core layer thicknesses >70 nm, representing a spectral tuning range of >50 nm over which we maintain excellent angular stability. The increase in transmission for increasing cavity thickness is directly related to reducing the parasitic absorption from the organic layer through uncoupled excitons. Above 70 nm, this parasitic absorption vanishes completely, and the remaining losses are due to (i) parasitic absorption in the metal layer and (ii) Fresnel-reflection at the substrate-air interface. Neither of these can be further reduced by a change in cavity thickness and neither is related to the presence of the organic material inside the cavity. This thus represents the best possible transmission in such a design.

Supplementary Figure 14 showcases the polarization-resolved transmission of the SubPc-based filter compared to a conventional MDM filter. While the conventional filter shows a strong angle-dependence and polarization-splitting at large angles, the polariton filter shows a stable spectrum for both polarizations. Due to the different interaction of the two polarizations with the mirror layers, both filters show a higher transmission in p-polarization compared to s-polarization at high angles.

Finally, dielectric thin film stacks offer the best optical performance for both conventional and polariton-based filters. The Optimized Polariton Filter 1 (OPF1) shows a measured peak transmission of 93%, with the remaining loss largely due to Fresnel reflections at the back substrate-air interface. Supplementary Figure 15 **a** shows that this reflection can be efficiently reduced by using a single or multilayer backside anti-reflection coating (ARC). Even the addition of just a single low-index film of MgF_2 increases the peak transmission to 96%, while a 9-layer ARC further enhances transmission to 98%. By simultaneously optimizing the ARC and the front-side filter, we can further enhance the performance as shown in Supplementary Fig. 15 **b**, leading to an improved design that exhibits >98% transmission in its passband for angles up to 40° and >93% for angles up to 60°, without any major spectral shift of the edge onset.

We are therefore confident that such designs will compare very well against any conventional high-performance multilayer stack and that they outperform current approaches toward angular stability. To quantify this further, Supplementary Figure 16 and Supplementary Table 2 compare the performance of our polariton filters to solutions discussed in the current scientific literature. We find that polariton filters reach or exceed the performance of current solutions while keeping design complexity and fabrication effort low, especially when compared to approaches requiring nano-scale patterning, such as plasmonics and metasurfaces. The cost of the common organic materials that we used to induce strong coupling is also much lower than that of high-index materials, which are the basis of the best performing current alternatives used in research and industry.

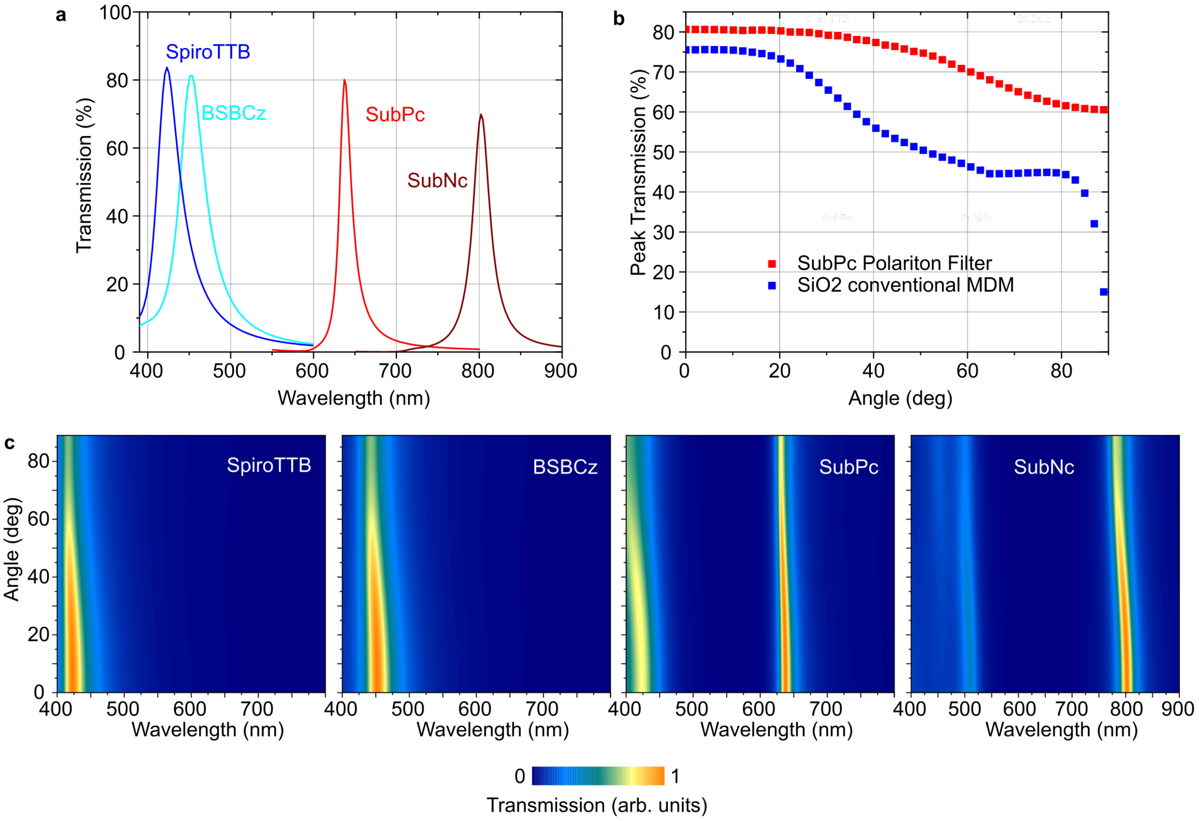

Supplementary Figure 11 Ag-Ag polariton filters with optimized peak transmission. a Simulated transmission spectra for a series of metal-organic-metal polariton filters tuned to different wavelengths and optimized for peak transmission. Data obtained by transfer matrix simulations, assuming silver films of 25 nm thickness and with ideal optical properties[3]. **b** Simulated peak transmission vs angle for a SubPc-based polariton filter and a conventional SiO_2-based MDM filter tuned to the same resonance wavelength at 0° angle of incidence. The polariton filter maintains a higher peak transmission at large angles even when taking the spectral red-shift of the conventional cavity into account. **c** Simulated angle-resolved transmission spectra for the filters shown in **a** and **b**.

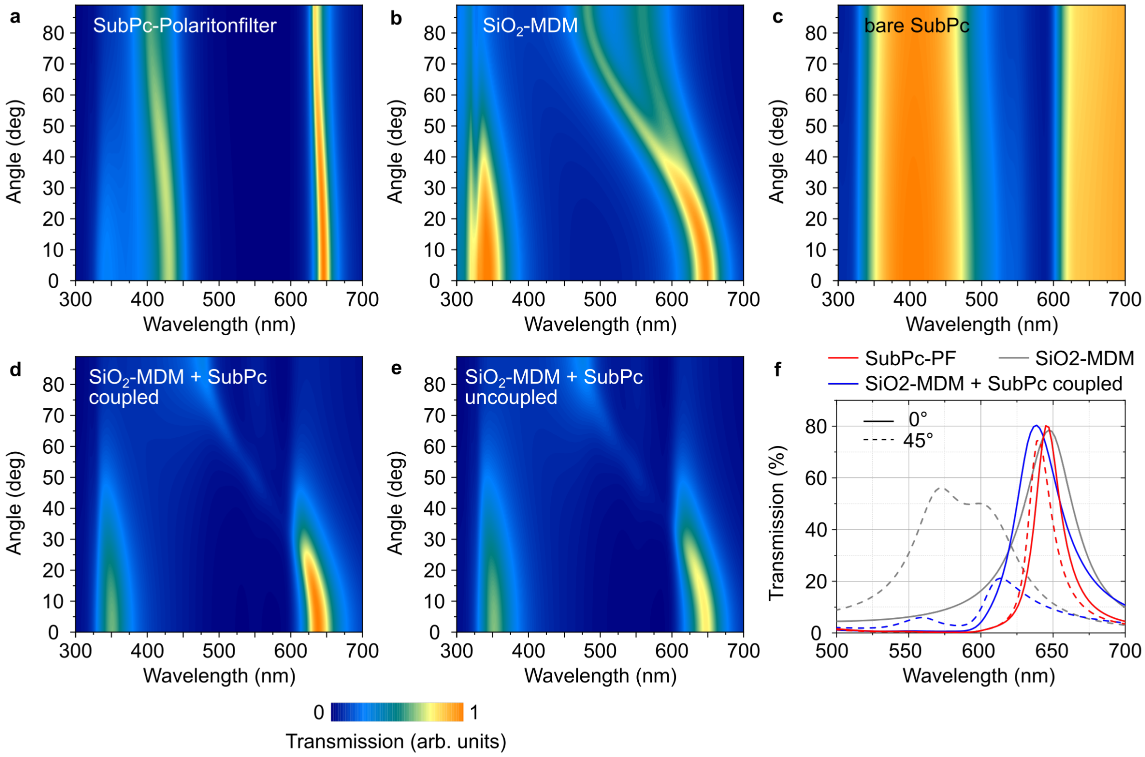

Supplementary Figure 12 Comparing polariton filters against MDM filters, absorptive filters and combined MDM / absorptive filters. a-e Angle-resolved transmission of **a** 25 nm Ag | SubPc | 25nm Ag polariton filter, **b** 25 nm Ag | SiO_2 | 25nm Ag MDM-filter, **c** 80 nm SubPc absorptive Filter, **d** 80nm SubPc | 25 nm Ag | SiO_2 | 25nm Ag combined absorptive MDM filter with both elements directly next to each other allowing for some degree of optical coupling between the layers, **e** 80nm SubPc | Substrate | 25 nm Ag | SubPc | 25nm Ag combined absorptive/MDM filter without optical coupling between the two elements. **f** Direct comparison of SubPc polariton filter (red), SiO_2-MDM (grey) and coupled SiO_2-MDM + SubPc (blue) at 0° (solid lines) and 45° (dashed lines) angle of incidence. The polariton filter acts fundamentally different to a combination of a conventional dielectric and absorptive filters, as the transmission is redirected towards the polariton branches instead of just being passively absorbed, resulting in a high and spectrally stable transmission at all angles.

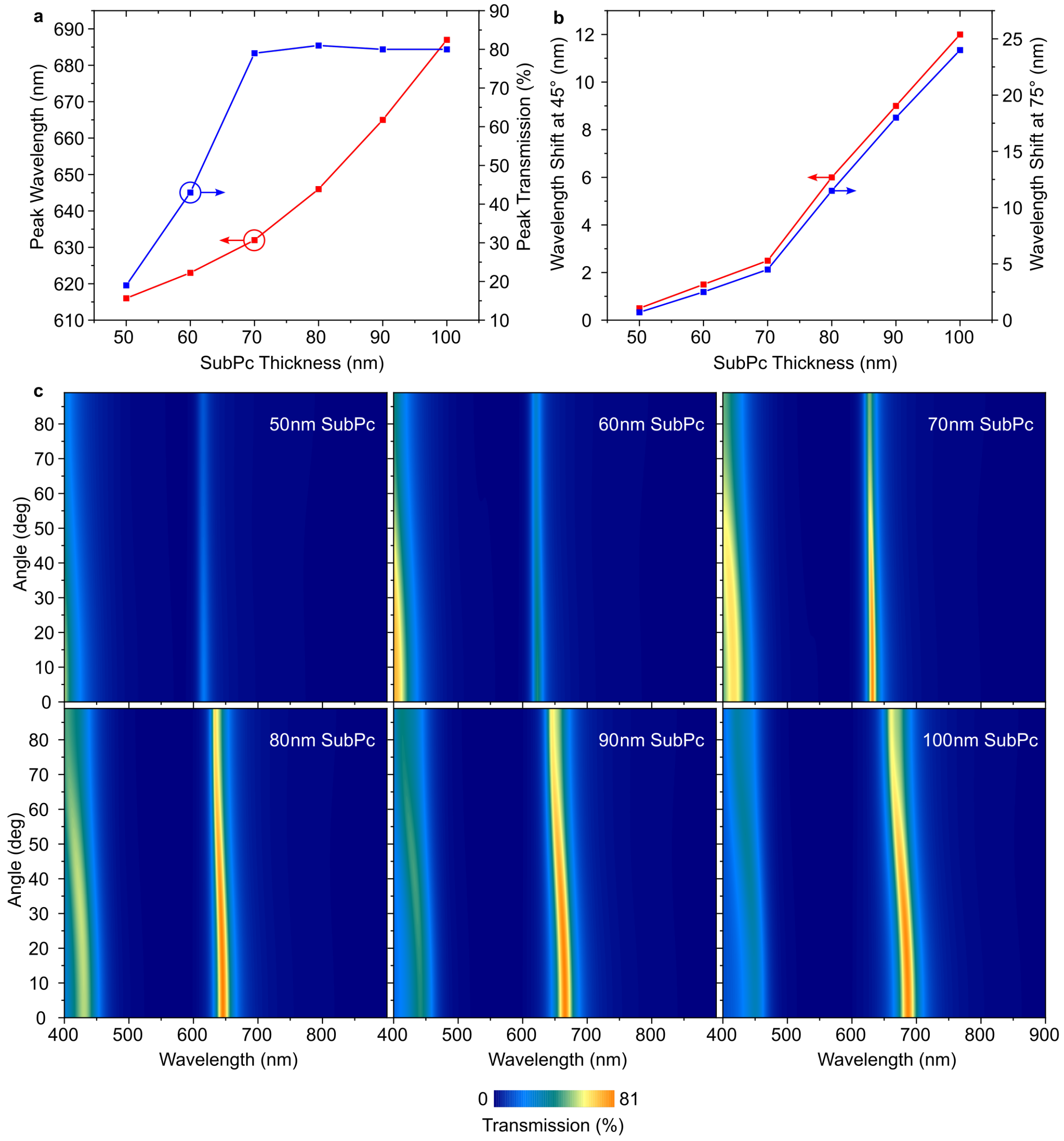

Supplementary Figure 13 Tunability of polariton filters. a Peak wavelength and peak transmission of a 25 nm Ag | SubPc | 25nm Ag polariton filter as a function of the thickness of the SubPc core. By merely changing the thickness of the SubPc layer and thus the detuning of the cavity photon with respect to the exciton, the transmission wavelength of the polariton filter can be tuned by >50 nm (and by >80 nm when accepting reduced performance); i.e. a single material can be used to cover a broad spectral range. **b** Shift of peak transmission wavelength at 45° and 75° angle of incidence as a function of SubPc thickness. Increasing the SubPc thickness leads to an increasing blue-shift of the lower polariton with angle. However, even at a thickness of 100 nm, the blueshift remains small in absolute terms (~12 nm at 45°, ~24 nm at 75°). **c** Simulated angle-resolved transmission spectra for the data presented in **a** and **b**.

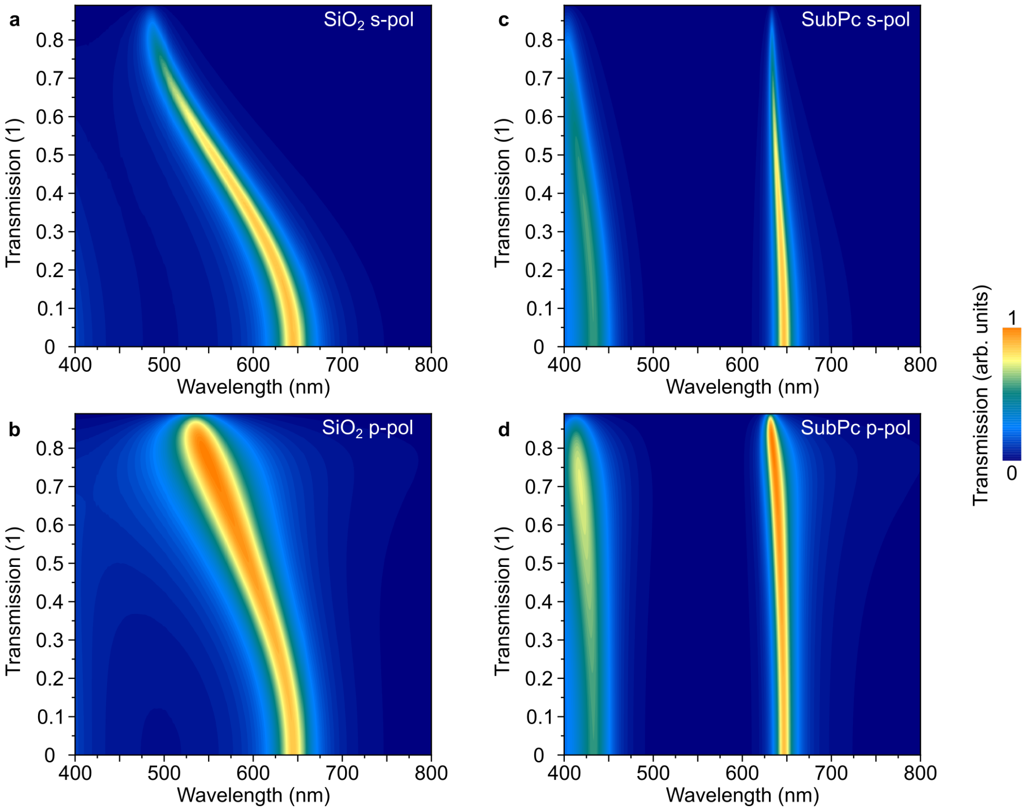

Supplementary Figure 14 Polarized transmission of polariton filters. a,**b** Transmission of a 25 nm Ag | 168 nm SiO_2 | 25nm Ag MDM filter in s- (**a**) and p-polarization (**b**). **c**,**d** Transmission of a 25 nm Ag | 80 nm SubPc | 25nm Ag polariton filter in s- (**c**) and p-polarization (**d**).

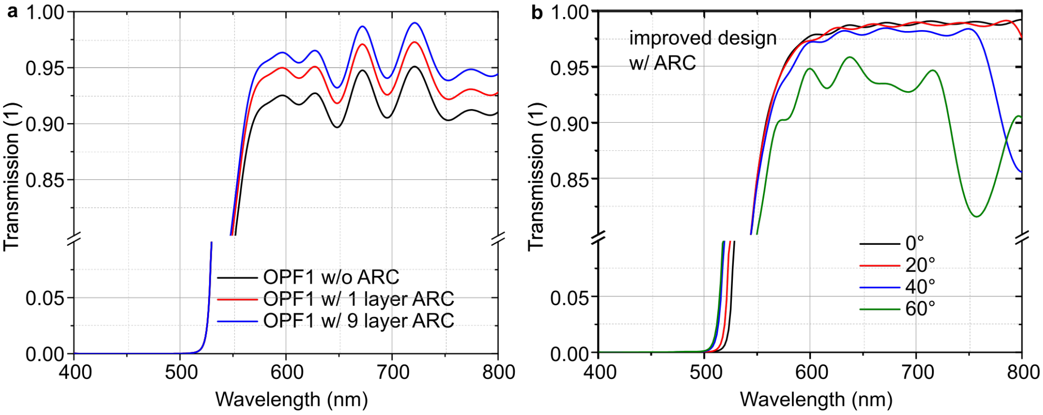

Supplementary Figure 15 High-performance design of a DBR-based longpass polariton filter. a Simulated transmission spectra of Optimized Polariton Filter 1 (OPF1) without anti-reflection coating (ARC, black), with a single layer MgF_2 backside ARC (red) and with a 9-layer backside ARC (blue). Using a single MgF_2 layer as ARC improves transmission by ~3%, using a more complex 9-layer ARC improves transmission by ~5%. **b** Improved design obtained by simultaneous optimization of frontside polariton filter and backside ARC coating. The improved filter shows a stable transmission of >98% for angles up to 40° and >93% up to 60°.

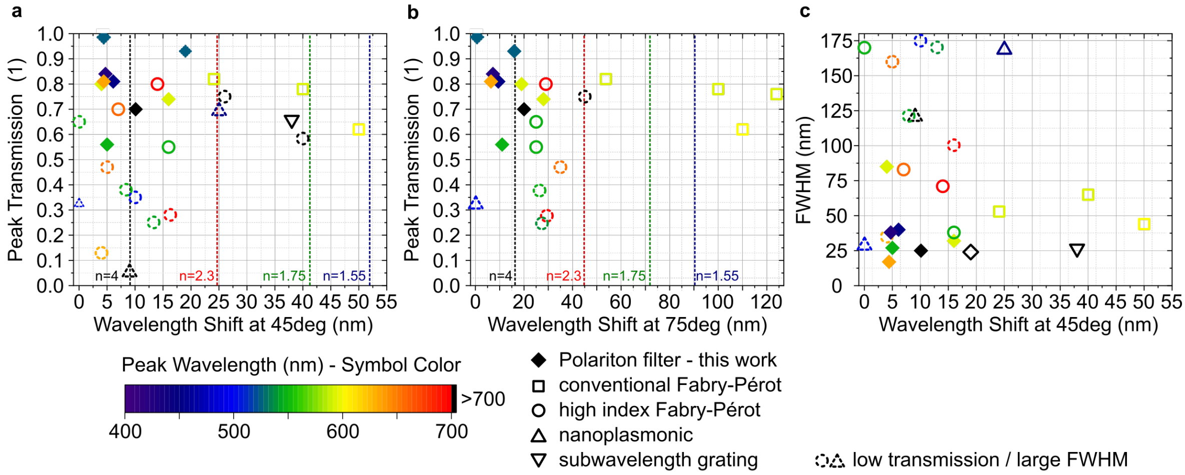

Supplementary Figure 16 Literature comparison of angle-stable bandpass transmission filters.

a Peak transmission of different filters vs wavelength shift at 45° angle of incidence. **b** Peak transmission of different filter vs wavelength shift at 75° angle of incidence. **c** Full width at half maximum of the main transmission line of different filters vs wavelength shift at 45° angle of incidence. Symbols are coded by colour (peak wavelength) and shape as indicated in the legend. Polariton filters are represented by filled symbols, all other filters are represented by open symbols.

Supplementary Table 2: Literature comparison of angle-stable bandpass transmission filters.

Filter-Type	Max T[1]	FWHM[2]	Δλ [3] at 45°	Δλ at 75°	λ [4] at 0°	complexity	Ref.
Unit	%	nm	nm	nm	nm		
**Polariton Filter - Experiment**							
C545T	56	27	5	11	545	low	this
C545T BP	80	85	4	19	590	low-med	this
OPF1	93	LP	19	16	525	medium	this
**Polariton Filter - Simulation**							
C545T	74	32	16	28	590	low	this
SubNc	70	25	10.1	20	802	low	this
SubPc	81	17	4.4	6.4	638	low	this
BSBCz	81	40	6.1	9.4	452	low	this
SpiroTTB	84	38	4.7	7.2	423	low	this
OPF1-ARC	98	LP	4.4	1.1	530	medium	this
**Conventional FP filter - Simulation**							
SiO2	76	45	61	124	590	low	this
Ta2O5	82	53	24.1	53.7	590	low	this
**Literature on angle-stable bandpass transmission filters**							
FP cavity	62	44	50	110	600	low	4
FP cavity	78	65	40	>100	600	low	5
HI-FP cavity	55	38	16	>25	550	medium	6
HI-FP cavity	75	190	26	45	950	medium	7
HI-FP cavity	70	83	7	-	660	medium	8
HI-FP cavity	35	175	10	-	500	medium	9
HI-FP cavity	65	170	0	25	550	medium	10
HI-FP cavity	25	170	13	27	540	medium	11
HI-FP cavity	28	100	16	29	720	medium	11
HI-FP cavity	38	121	8	26	545	medium	12
HI-FP cavity	58	200	40	-	900	medium	13
HI-lossy FP	47	160	5	35	650	low	14
HI-DBR-metal	13	35	4	-	635	high	15
HI-DBR	80	71	14	29	700	med-high	16
Polaritonic cavity	low	24	19	>35	900	low	17
DBR-plasmonic	33	30	0	0	500	high	18
Nanorod	6	122	9	-	1400	high	19
Plasmonic array	70	170	25	-	450	high	20
DBR-grating	65	25	38	-	800	high	21

[1] maximum transmission of the main transmission line
[2] full width at half maximum of the main transmission line
[3] wavelength shift of the main transmission line at indicated angle of incidence
[4] central wavelength of the main transmission line

Abbreviations:

OPF1	…	Optimized Polariton Filter 1	HI	…	high index of refraction
LP	…	Longpass	DBR	…	distributed Bragg reflector
FP	…	Fabry-Pérot	-	…	data not provided in publication

Supplementary Note 4

The monolithic filter-photodiode stack is based on two stacked coupled cavities, namely the filter and the diode, with a shared central mirror/anode. Typically, such systems would need to be optically decoupled to avoid interaction between these cavities. Recently, such an organic platform was demonstrated by fabricating filter and diode layers on opposite sides of a mm-thick glass substrate[22]. In the case of polariton filters however, the strong absorption present in the filter cavity efficiently suppresses the formation of coupled optical resonances if all layer thicknesses are optimized.

The current density-voltage (jV) measurements, depicted in Supplementary Fig. 17, confirm robust diode operation both with and without the additional filter, showing a slightly increased reverse-bias current density for the filtered diode with respect to the reference, indicating that the filter might introduce additional shunts. For both the filtered and unfiltered diodes, we estimate specific detectivities of up to the order of 10^{12} Jones, in agreement with previous results on similar diodes[23,24]. To estimate detectivity, we took into account the measured peak external quantum efficiency (EQE) of 18% and describe the electrical noise as sum of thermal and shot noise[25] at zero bias voltage (a full determination of the noise is beyond the scope of the current study).

To accurately model the absorption in the combined filter-photodiode stack, we perform electric field simulations of the device (Supplementary Fig. 18**a**). The polariton formation in the metal-C545T-metal cavity efficiently filters the light entering the active layer of the photodiode (red shaded area). Using an additional C545T absorption filter can further enhance the spectral selectivity and reduce unwanted signal between 400 nm and 500 nm (Supplementary Fig. 18**b**).

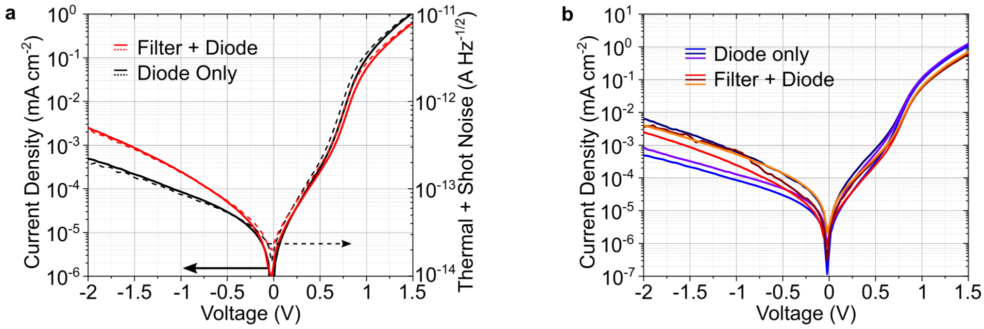

Supplementary Figure 17: Current-density – voltage characteristics of filtered and reference photodiodes. a Current density – voltage (solid lines) behaviour and estimated thermal and shot noise current (dashed lines) for the filtered (red lines) and unfiltered (black lines) photodiode. The noise current at 0 V is not influenced by the addition of the polariton filter. **b** Current-density - voltage characteristics for three reference photodiodes and three photodiodes comprising a polariton filter, respectively. The diode characteristics are reproducible for different samples, with the filtered diodes showing a slightly increased reverse current, likely due to higher roughness.

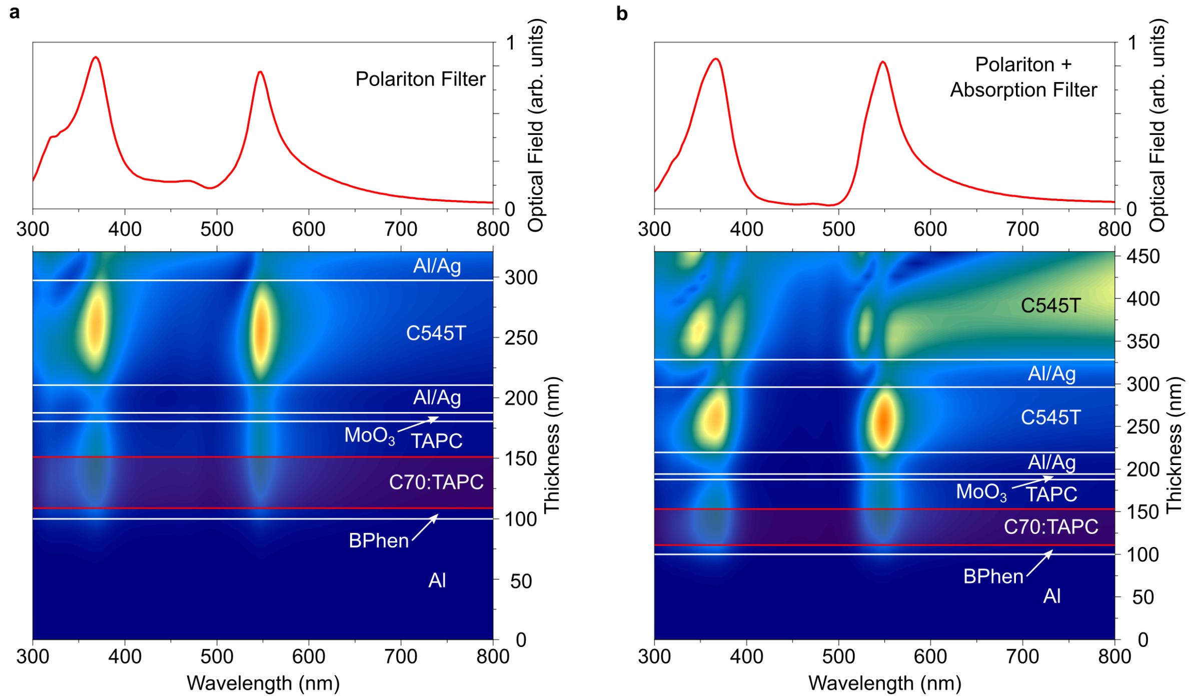

Supplementary Figure 18: Simulations of optical field in polariton-filter-photodiode. a Simulated electric field of the photodiode comprising a polariton filter with the structure 1 nm Al | 25 nm Ag | 80 nm C545T | 1 nm Al | 25 nm Ag | 7 nm MoO_3 | 30 nm TAPC | 40 nm C70:TAPC (5wt%) | 12 nm BPhen | 100 nm Al. The profile on top shows the integrated electric field in the photoactive area of the device (C70:TAPC, region marked in red). **b** Simulated electric field of the filtered diode shown in **a** with an additional 150 nm C545T absorption layer on top. This additional absorptive filter reduces the diode signal in the blocking region between 400 nm and 500 nm.

Supplementary References

1. Mischok, A. *et al.* Controlling Tamm Plasmons for Organic Narrowband Near-Infrared Photodetectors. *ACS Photonics* **4**, 2228–2234 (2017).
2. Zhang, C., Ji, C., Park, Y. & Guo, L. J. Thin-Metal-Film-Based Transparent Conductors: Material Preparation, Optical Design, and Device Applications. *Adv. Opt. Mater.* **9**, 2001298 (2021).
3. McPeak, K. M. *et al.* Plasmonic Films Can Easily Be Better: Rules and Recipes. *ACS Photonics* **2**, 326–333 (2015).
4. Li, Z., Butun, S. & Aydin, K. Large-area, Lithography-free super absorbers and color filters at visible frequencies using ultrathin metallic films. *ACS Photonics* **2**, 183–188 (2015).
5. Williams, C., Gordon, G. S. D., Wilkinson, T. D. & Bohndiek, S. E. Grayscale-to-Color: Scalable Fabrication of Custom Multispectral Filter Arrays. *ACS Photonics* **6**, 3132–3141 (2019).
6. Lee, K.-T., Han, S. Y., Li, Z., Baac, H. W. & Park, H. J. Flexible High-Color-Purity Structural Color Filters Based on a Higher-Order Optical Resonance Suppression. *Sci. Rep.* **9**, 14917 (2019).
7. Jen, Y.-J. & Lin, M.-J. Design and Fabrication of a Narrow Bandpass Filter with Low Dependence on Angle of Incidence. *Coatings* **8**, 231 (2018).

8. Song, C., Feng, Y., Bao, Z., Liu, G. & Wang, J. Angle-Insensitive Color Filters Based on Multilayer Ultrathin-Film Structures. *Plasmonics* **15**, 255–261 (2020).

9. Mao, K. *et al.* Angle Insensitive Color Filters in Transmission Covering the Visible Region. *Sci. Rep.* **6**, 19289 (2016).

10. Park, C.-S., Shrestha, V. R., Lee, S.-S., Kim, E.-S. & Choi, D.-Y. Omnidirectional color filters capitalizing on a nano-resonator of Ag-TiO2-Ag integrated with a phase compensating dielectric overlay. *Sci. Rep.* **5**, 8467 (2015).

11. Lee, J. Y., Lee, K.-T., Seo, S. & Guo, L. J. Decorative power generating panels creating angle insensitive transmissive colors. *Sci. Rep.* **4**, 4192 (2014).

12. Guo, L. J. & Xu, T. Spectrum filtering for visual displays and imaging having minimal angle dependence. (2012).

13. Noh, T.-H., Yoon, Y.-T., Lee, S.-S., Choi, D.-Y. & Lim, S.-C. Highly Angle-tolerant Spectral Filter Based on an Etalon Resonator Incorporating a High Index Cavity. *J. Opt. Soc. Korea* **16**, 299–304 (2012).

14. Lee, K.-T. T., Seo, S., Yong Lee, J. & Jay Guo, L. Ultrathin metal-semiconductor-metal resonator for angle invariant visible band transmission filters. *Appl. Phys. Lett.* **104**, 231112 (2014).

15. Tessler, N., Burns, S., Becker, H. & Friend, R. H. Suppressed angular color dispersion in planar microcavities. *Appl. Phys. Lett.* **70**, 556–558 (1997).

16. Shrestha, V. R., Lee, S.-S., Kim, E.-S. & Choi, D.-Y. Non-iridescent Transmissive Structural Color Filter Featuring Highly Efficient Transmission and High Excitation Purity. *Sci. Rep.* **4**, 4921 (2014).

17. Gambino, S. *et al.* Exploring Light–Matter Interaction Phenomena under Ultrastrong Coupling Regime. *ACS Photonics* **1**, 1042–1048 (2014).

18. He, X. *et al.* A single sensor based multispectral imaging camera using a narrow spectral band color mosaic integrated on the monochrome CMOS image sensor. *APL Photonics* **5**, (2020).

19. Wells, B. M., Lotti, F., Nasir, M. E., Zayats, A. V. & Podolskiy, V. A. Angle-insensitive plasmonic nanorod metamaterial-based band-pass optical filters. *Opt. Express* **29**, 11562 (2021).

20. Yang, C. *et al.* Angle Robust Reflection/Transmission Plasmonic Filters Using Ultrathin Metal Patch Array. *Adv. Opt. Mater.* **4**, 1981–1986 (2016).

21. Xiang, J., Song, M., Zhang, Y., Kruschwitz, J. & Cardenas, J. Ultrabroadband, High Color Purity Multispectral Color Filter Arrays. *ACS Photonics* **11**, 1163–1172 (2024).

22. Xing, S. *et al.* Miniaturized VIS-NIR Spectrometers Based on Narrowband and Tunable Transmission Cavity Organic Photodetectors with Ultrahigh Specific Detectivity above 10 14 Jones. *Adv. Mater.* **33**, 2102967 (2021).

23. Shekhar, H. *et al.* Hybrid image sensor of small molecule organic photodiode on CMOS – Integration and characterization. *Sci. Rep.* **10**, 7594 (2020).

24. Yang, D. & Ma, D. 1,1-Bis[(di-4-tolylamino)phenyl]cyclohexane for fast response organic photodetectors with high external efficiency and low leakage current. *J. Mater. Chem. C* **1**, 2054 (2013).

25. Siegmund, B. *et al.* Organic narrowband near-infrared photodetectors based on intermolecular charge-transfer absorption. *Nat. Commun.* **8**, 15421 (2017).
